# Uveitis as a Result of MAP Kinase Pathway Inhibition

**DOI:** 10.1159/000357060

**Published:** 2013-11-30

**Authors:** Lavnish Joshi, Andreas Karydis, Maria Gemenetzi, Emily H. Shao, Simon R.J. Taylor

**Affiliations:** ^a^Royal Surrey County Hospital NHS Foundation Trust, Guildford, Surrey, London, UK; ^b^Faculty of Medicine, Imperial College London, London, UK

**Keywords:** Dabrafenib, Trametinib, Uveitis, Melanoma

## Abstract

We report the case of a patient treated with dabrafenib and trametinib (mitogen-activated protein kinase pathway inhibitors) for stage 3b cutaneous melanoma who developed bilateral uveitis. Although there have been reports of ocular side effects with this class of drugs, uveitis has not been previously reported to the best of our knowledge. This case indicates the wide range of side effects that can be seen with the newer targeted biological therapies.

## Introduction

Molecularly targeted therapies are potentially more specific with fewer toxic side effects than many traditional chemotherapeutic agents used in the treatment of cancer [1]. Nevertheless, they can be associated with a variety of toxicities. We report a case of a patient treated with dabrafenib and trametinib [mitogen-activated protein kinase (MAP kinase) pathway inhibitors] for stage 3b cutaneous melanoma who developed bilateral uveitis while on therapy. To the best of our knowledge, although there have been reports of ocular side effects with this class of drugs, uveitis has not been previously reported [2].

## Case Report

A 64-year-old male underwent adjuvant treatment with oral dabrafenib 150 mg b.d. and oral trametinib 2 mg o.d. for a cutaneous nodular ulcerating melanoma under his left breast, as part of the COMBI-AD clinical trial (a trial of dabrafenib and trametinib after surgery to remove melanoma). He previously underwent surgical resection of the tumour (Breslow thickness 2.15 mm, BRAF gene positive, stage 3b) and also had positive sentinel node biopsies. Ocular examinations form part of the protocol of this trial, owing to previous associations between these agents and retinal vein occlusion and central serous chorioretinopathy [3].

**Fig. 1:**
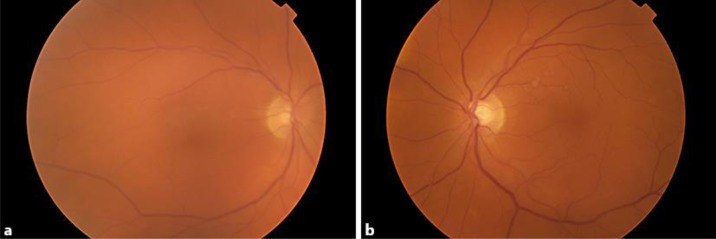
Fundus photographs demonstrating 0.5+ vitreous haze in both eyes associated with vitreous snowballs anterior to the superotemporal retinal arcade in the left eye. All findings resolved spontaneously without treatment.

**Fig. 2:**
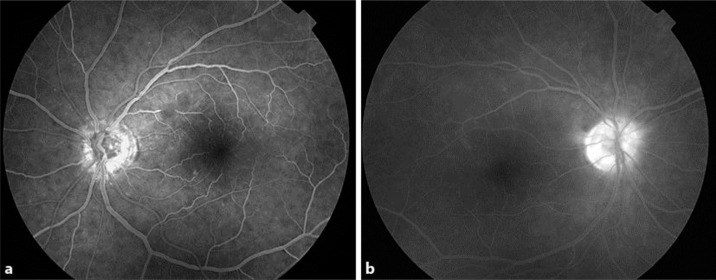
Fluorescein angiography demonstrating early patchy choroidal hyperfluorescence in **a**, and late optic disc leakage in **b**.

At his initial screening visit, ocular examination was unremarkable, apart from an area of peripheral retinal degeneration in his right eye. Two weeks after starting the trial medications, he experienced pyrexia and malaise. His medications were temporarily stopped and he was started on ibuprofen; an ocular examination at this stage also proved normal. One week later, the decision was made to stop the trial medications permanently. However, he developed floaters in the left eye after a few days, and an ocular examination revealed vitritis in both eyes (2+ vitreous cells, 0.5+ vitreous haze), with vitreous snowballs in the left eye anterior to the superotemporal retinal arcade (fig. 1). His visual acuity remained unaffected at 6/6. Fluorescein angiography indicated early patchy choroidal hyperfluorescence in both eyes together with late optic disc leakage (fig. 2).

Endogenous endophthalmitis was considered unlikely owing to the absence of focal chorioretinal involvement, the lack of significant immunosuppression and the absence of any predisposing invasive procedures within the recent past. A presumptive diagnosis of drug-induced inflammation was made, and it was elected to observe him without additional treatment. Within 6 weeks, all signs of ocular inflammation had resolved without treatment and without sequelae.

## Discussion

Dabrafenib and trametinib are inhibitors of the MAP kinase pathway, a pathway that ultimately leads to cellular proliferation. MAP kinase pathway inhibitors have been under investigation in the treatment of a number of tumour types, including melanoma, and the aim of combining several MAP kinase inhibitors in a patient is to improve response rates, to delay resistance and to reduce drug toxicity, since lower doses of each drug can be used [4].

Some drugs in this class have been associated with ocular side effects in up to 27% of patients [3]. The most serious reported side effect is retinal vein occlusion [5], but uveitis has not been previously reported to the best of our knowledge. The mechanism behind these ocular side effects remains unclear, but it has been suggested that MAP kinase inhibition can lead to an inflammatory response with consequent breakdown of the blood-retinal barrier [6]. This could potentially compromise ocular immune privilege, generating autoimmune uveitis, thus providing an explanation for the findings in our case [7, 8].

## Disclosure Statement

The authors have no conflicts of interest.
